# Programmatic Precision Oncology Decision Support for Patients With Gastrointestinal Cancer

**DOI:** 10.1200/PO.22.00342

**Published:** 2023-01-12

**Authors:** Rachel B. Keller, Tali Mazor, Lynette Sholl, Andrew J. Aguirre, Harshabad Singh, Nilay Sethi, Adam Bass, Ankur K. Nagaraja, Lauren K. Brais, Emma Hill, Connor Hennessey, Margaret Cusick, Catherine Del Vecchio Fitz, Zachary Zwiesler, Ethan Siegel, Andrea Ovalle, Pavel Trukhanov, Jason Hansel, Geoffrey I. Shapiro, Thomas A. Abrams, Leah H. Biller, Jennifer A. Chan, James M. Cleary, Steven M. Corsello, Andrea C. Enzinger, Peter C. Enzinger, Robert J. Mayer, Nadine J. McCleary, Jeffrey A. Meyerhardt, Kimmie Ng, Anuj K. Patel, Kimberley J. Perez, Osama E. Rahma, Douglas A. Rubinson, Jeffrey S. Wisch, Matthew B. Yurgelun, Michael J. Hassett, Laura MacConaill, Deborah Schrag, Ethan Cerami, Brian M. Wolpin, Jonathan A. Nowak, Marios Giannakis

**Affiliations:** ^1^ Department of Medical Oncology, Dana-Farber Cancer Institute & Harvard Medical School, Boston, MA; ^2^ Department of Data Science, Dana-Farber Cancer Institute, Boston, MA; ^3^ Center for Advanced Molecular Diagnostics, Brigham & Women's Hospital & Harvard Medical School, Boston, MA; ^4^ Broad Institute of Harvard and MIT, Cambridge, MA

## Abstract

With the growing number of available targeted therapeutics and molecular biomarkers, the optimal care of patients with cancer now depends on a comprehensive understanding of the rapidly evolving landscape of precision oncology, which can be challenging for oncologists to navigate alone.

**METHODS:**

We developed and implemented a precision oncology decision support system, GI TARGET, (Gastrointestinal Treatment Assistance Regarding Genomic Evaluation of Tumors) within the Gastrointestinal Cancer Center at the Dana-Farber Cancer Institute. With a multidisciplinary team, we systematically reviewed tumor molecular profiling for GI tumors and provided molecularly informed clinical recommendations, which included identifying appropriate clinical trials aided by the computational matching platform MatchMiner, suggesting targeted therapy options on or off the US Food and Drug Administration–approved label, and consideration of additional or orthogonal molecular testing.

**RESULTS:**

We reviewed genomic data and provided clinical recommendations for 506 patients with GI cancer who underwent tumor molecular profiling between January and June 2019 and determined follow-up using the electronic health record. Summary reports were provided to 19 medical oncologists for patients with colorectal (n = 198, 39%), pancreatic (n = 124, 24%), esophagogastric (n = 67, 13%), biliary (n = 40, 8%), and other GI cancers. We recommended ≥ 1 precision medicine clinical trial for 80% (406 of 506) of patients, leading to 24 enrollments. We recommended on-label and off-label targeted therapies for 6% (28 of 506) and 25% (125 of 506) of patients, respectively. Recommendations for additional or orthogonal testing were made for 42% (211 of 506) of patients.

**CONCLUSION:**

The integration of precision medicine in routine cancer care through a dedicated multidisciplinary molecular tumor board is scalable and sustainable, and implementation of precision oncology recommendations has clinical utility for patients with cancer.

## INTRODUCTION

In parallel with a deepening understanding of the molecular landscapes of cancer, the standards of care in clinical oncology are also evolving. Cancers are driven by heterogeneous molecular alterations across tissues of origin, among individuals with the same cancer diagnosis^1^ and even among subclones within a single tumor.^2^ Precision oncology involves identifying the molecular changes that drive malignancy in an individual tumor and targeting these oncogenic alterations, a strategy that can lead to different therapeutic approaches in patients with the same cancer diagnosis. Although achieving clinical responses is of paramount interest, targeted therapies may also ameliorate some of the toxicities and morbidity associated with cytotoxic cancer treatment. Accordingly, many institutions implement precision oncology programs and practices.^3-10^

**CONTEXT**

**Key Objective**
The increasing complexity of the precision oncology landscape requires the development of programs and resources to support informed clinical decision making. We describe our experience in developing a large-scale multidisciplinary precision oncology decision support program within the Gastrointestinal Cancer Center at the Dana-Farber Cancer Institute.
**Knowledge Generated**
In providing molecularly informed recommendations for hundreds of patients with gastrointestinal cancer on the basis of review of tumor molecular profiling, we demonstrate the scalability and sustainability of precision oncology through implementation of innovative workflows. In a retrospective analysis of 500+ cases, we show the clinical utility of our program with impacts on clinical trial enrollment, germline testing, and other provider actions.
**Relevance**
Through the systematic and critical evaluation of a large-scale precision oncology effort, our experience has the potential to inform similar efforts, in both academic and community settings, and highlights the importance of multidisciplinary molecular tumor boards in the current practice of oncology.

As part of the PROFILE initiative at Dana-Farber/Brigham and Women's Cancer Center and Dana-Farber/Boston Children's Cancer and Blood Disorders Center, more than 35,000 patient tumors have been characterized using the targeted next-generation sequencing platform OncoPanel since 2013.^11,12^ The current version of OncoPanel (v3.1) provides coverage of 447 cancer-associated genes allowing for the identification of single-nucleotide variants/insertions/deletions and copy number variants as well as structural rearrangement variants of select genes (Supplemental Table 1 in Data Supplement). OncoPanel has been used in routine clinical practice for some tumor types since 2014. However, interpretation and application of genomic testing for real-time clinical decision making is complex. A working knowledge of cancer genomics and the capabilities and limitations of current molecular testing methods is necessary to capitalize on the information gleaned from molecular profiling. Furthermore, the fast-moving landscape of biomarker-therapy associations and the constantly evolving portfolio of precision medicine clinical trials can confound the choice of targeted therapy options. The GI TARGET (Gastrointestinal Treatment Assistance Regarding Genomic Evaluation of Tumors) program was developed to provide precision oncology decision support for oncologists within the Dana-Farber Cancer Institute (DFCI) Gastrointestinal Cancer Center (GCC) by offering expert-led guidance on the interpretation and implementation of OncoPanel and alternative tumor molecular profiling offered by commercial laboratories.

With the GI TARGET program, we hoped to (1) reduce the burden on oncologists by providing consistent expert-led review of tumor profiling results, (2) assist in identifying appropriate targeted therapy options for patients, (3) support clinical trial enrollment using computational matching to ongoing studies guided by tumor molecular data, and (4) provide an example framework for how precision oncology can be incorporated into routine clinical cancer care, which may help guide efforts in diverse settings, eg, community clinics and hospitals where the majority of patients with cancer in the United States receive care. To assess the feasibility and impact of the GI TARGET program, we analyzed the genomics review process and clinical follow-up for 506 patients who underwent tumor molecular profiling between January and June 2019.

## METHODS

### Program Development

The GI TARGET program is a collaboration between the DFCI GCC, the Brigham & Women's Hospital Center for Advanced Molecular Diagnostics, and the DFCI Knowledge Systems Group, developers of the clinical trial matching platform MatchMiner.^13^ MatchMiner is an automated platform that allows for computational matching of patients to precision medicine clinical trials that are open to accrual at DFCI.^13^ MatchMiner is integrated with OncoPanel data such that molecular features detected in patient tumors can be compared with manually curated genomic eligibility criteria. As of the writing of this article, there were more than 400 precision medicine clinical trials curated in MatchMiner. Although OncoPanel reports genomic alterations with reference to therapeutic actionability, clinical trial matching via MatchMiner is a value-added feature of the GI TARGET report.

In January 2019, after a 12-month development phase, we expanded the GI TARGET program to the patients of 19 GCC medical oncologists. The intended patients for GI TARGET review were those with metastatic and/or recurrent disease who required additional treatment options beyond standard-of-care therapy; however, as review was systematically performed for all patients with GI cancer with recent OncoPanel results, patients with localized disease were also included. In addition, patients with previously resulted OncoPanel or alternative tumor profiling (eg, from commercial laboratories) were also reviewed at the request of the primary oncologist.

#### GI TARGET genomic resources and automation.

To enable systematic review of tumor profiling results, it was necessary to develop resources for extraction of pertinent data from OncoPanel and reassembly into a clear and concise format for presentation to providers. We therefore developed several knowledgebases (GI KBs) to facilitate the collection and curation of relevant GI cancer genomic and therapeutic information. With GI TARGET, our goal was to highlight actionable alterations detected on OncoPanel. The GI KBs (GI KB1—GI cancer genes of interest, GI KB2—single-nucleotide variants/insertions/deletions database, GI KB3—structural rearrangement variant database, and GI KB4—targeted therapy database) allowed for identification of alterations classified as oncogenic/likely oncogenic in genes that are clinically relevant or actionable in the context of GI cancers. A detailed description of the development of the GI KBs, including discussion of discrepancies between OncoPanel and GI TARGET variant interpretation, is provided in the Data Supplement. In addition to the GI KBs, we built two automated services to extend the existing MatchMiner infrastructure in support of GI TARGET to facilitate case review and report generation (Supplemental Figure 1, Data Supplement).

#### Review workflows.

**FIG 1.**
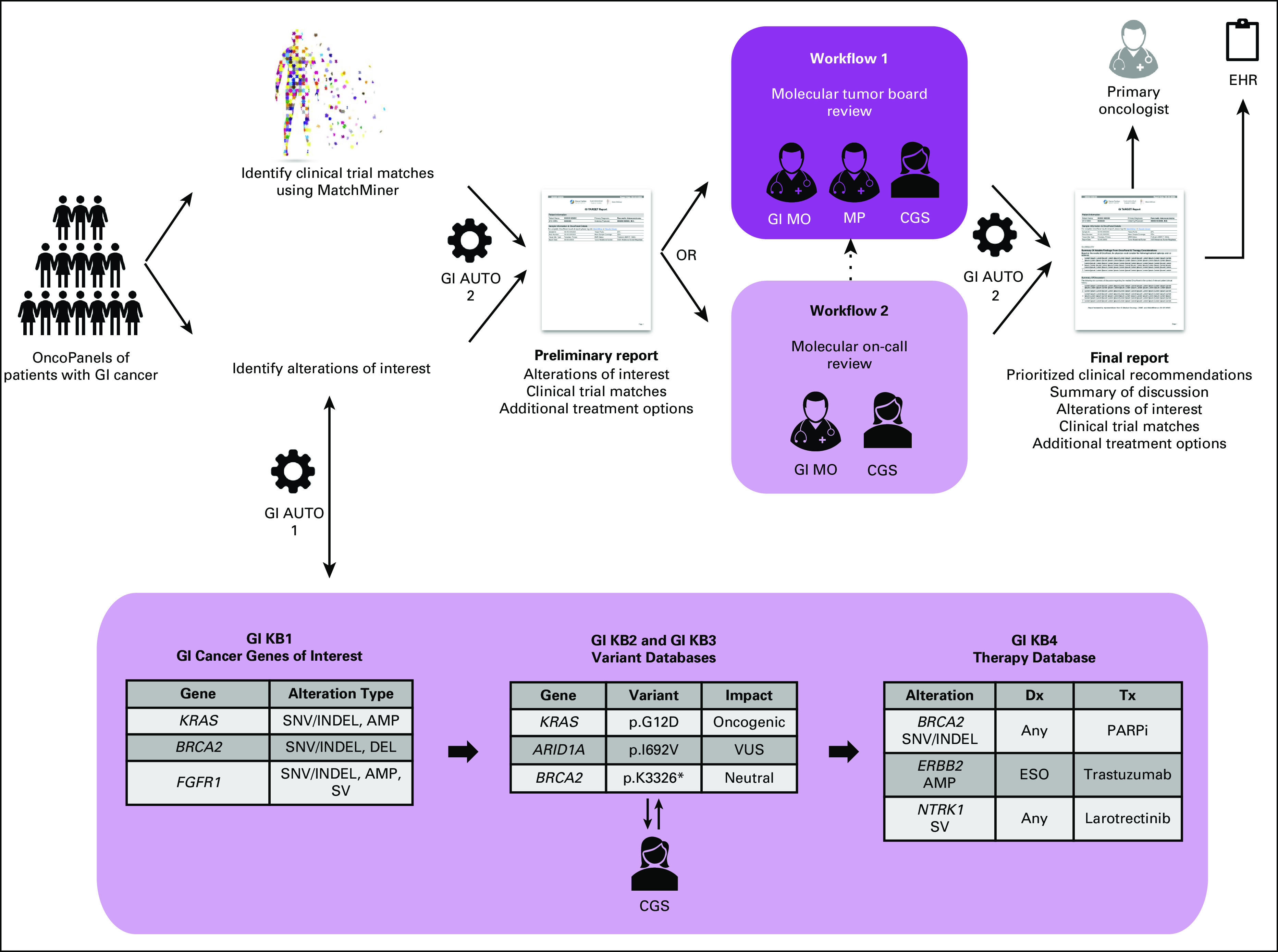
GI TARGET program workflow: Genomic information is extracted from OncoPanels of patients with GI cancer and organized into a series of GI KBs to facilitate interpretation and review. A preliminary report is generated, which summarizes alterations of interest and current clinical trial options identified through MatchMiner. This information is reviewed in the context of relevant clinicopathologic information either through multidisciplinary molecular tumor board discussion or offline through molecular on-call. Consensus clinical recommendations and GI TARGET team discussion are summarized in the GI TARGET final report, which is e-mailed to the primary oncologist and uploaded to the EHR. AMP, amplification; CGS, clinical genomic scientist; Dx, diagnosis; EHR, electronic health record; GI AUTO, GI automated service; GI MO, GI medical oncologist; GI TARGET, Gastrointestinal Treatment Assistance Regarding Genomic Evaluation of Tumors; INDEL, insertion/deletion; KB, knowledgebase; MP, molecular pathologist; SNV, single nucleotide variant; SV, structural variant; Tx, treatment; VUS, variant of unknown significance.

**FIG 2.**
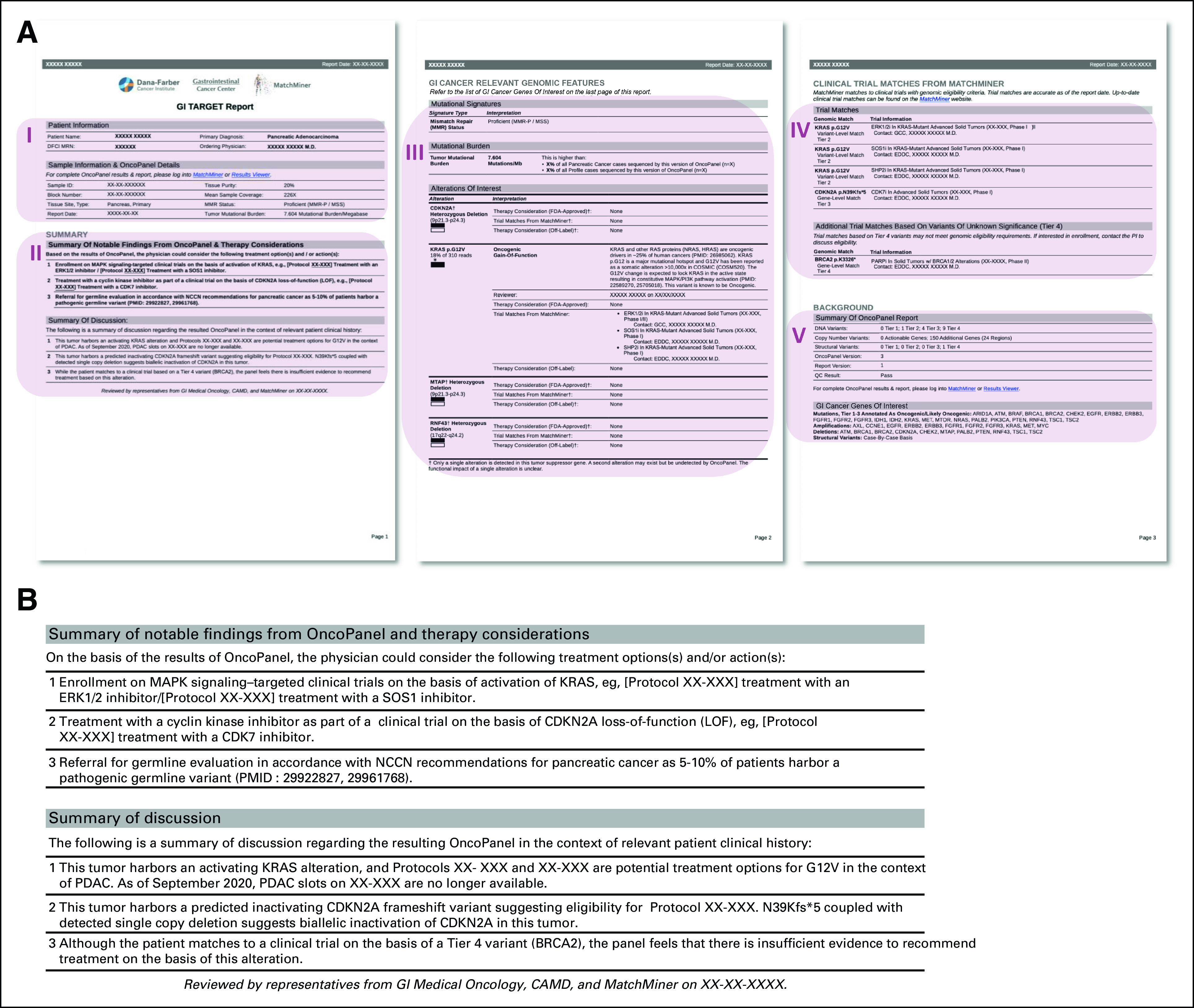
GI TARGET report content: (A) the PDF report includes (I) patient and sample information, (II) report summary, (III) GI cancer relevant genomic features, (IV) clinical trial matches from MatchMiner, and (V) additional report information. (II) is the most important section of the report and includes the "Summary of notable findings", which is a prioritized list of action items on the basis of integrated review of OncoPanel results in the context of relevant clinicopathologic information and additional commentary from the GI TARGET team that is useful for the primary oncologist. The "Summary of discussion" includes more detailed information expanding on the above recommendations. (IV) displays all clinical trial matches from MatchMiner on the basis of tier 1-3 SNV/INDELs, tier 1-3 SVs, and CNVs regardless of whether they are found in a gene listed in GI KB1 and, in the case of SNV/INDELs and SV, regardless of the interpretation (ie, oncogenic/likely oncogenic/VUS/neutral). Tier 4 SNV/INDELs are omitted from the GI TARGET report unless they are the basis of clinical trial matches, in which case they appear in a separate table titled "Additional Trial Matches Based on Variants of Unknown Significance." (B) Example of GI TARGET summary of notable findings from a pancreatic cancer case. CNVs, copy number variants; GI TARGET, Gastrointestinal Treatment Assistance Regarding Genomic Evaluation of Tumors; KB, knowledgebase; SNV/INDELs, single nucleotide variants/insertions/deletions; SV, structural rearrangement variants; VUS, variant of unknown significance.

To accommodate case volume, we established two parallel workflows for case review (Fig 1). Workflow 1 involved a weekly molecular tumor board (MTB) consisting of representatives from GI medical oncology (GCC), molecular pathology (Center for Advanced Molecular Diagnostics), the MatchMiner development team, and a dedicated Clinical Genomic Scientist (CGS). This forum was intended for complex cases such as tumors with many actionable alterations (ie, predictive of a specific therapeutic or other clinical intervention) for which prioritization of therapies warranted group discussion and cases where input regarding molecular/surgical pathology was desirable. Workflow 2, Molecular On-Call, involved review by a GCC medical oncologist with expertise in genomics in collaboration with the CGS. In this on-call system, a single oncologist was responsible for case review each week. This workflow was used to review the majority of straightforward cases, such as tumors with typical molecular profiles and tumors with few or no actionable alterations. At the reviewing oncologist's discretion, cases requiring additional input could undergo review at a subsequent weekly MTB. Experience from the GI TARGET development phase (January-June 2018), during which all cases were reviewed in a weekly MTB, guided decisions to triage to either workflow. Implementation of these workflows required the hiring of a 1.0 Full-Time Equivalent PhD-level CGS. The CGS was responsible for triaging the weekly caseload (ie, deciding which cases would be reviewed in each workflow), variant interpretation, initial review of cases and preparation of materials to expedite the review process, and compilation of finalized reports on the basis of recommendations and comments from reviewers as well as overall program management. Both workflows directly informed clinical recommendations that were detailed within a PDF report, which was e-mailed directly to the primary oncologist and made available in the electronic health record (EHR). An example GI TARGET report is shown in Figure 2A, and a detailed description of report content is provided in the Data Supplement.

### Program Assessment

#### Retrospective cohort.

For the retrospective analysis of the GI TARGET program, we assessed review of tumor profiling during the 6-month period from January to June 2019. All patients had consented to institutional review board–approved protocols at DFCI permitting access to their clinical and genomic data. A total of 539 patients with GI cancer underwent OncoPanel testing during this period. 547 OncoPanels from 539 patients, in addition to three patients with commercial molecular testing, entered the GI TARGET workflow for a total of 550 tumor profiling results from 542 patients. GI TARGET review was ultimately performed on 510 tumor profiling results from 506 patients.

#### Clinical follow-up assessment.

To assess primary oncologist action on the basis of recommendations made in GI TARGET reports, we collected clinical data from patient EHRs using a standardized data capture approach (PRISSMM)^14^ organized using a REDCap database.^15^ Follow-up data were collected from June 1, 2020, to November 17, 2020, and analyzed for the time period between the GI TARGET report date and the date of the last clinical visit at DFCI for each patient (range 1 day-23.2 months). Clinical follow-up data were assessable (ie, GI TARGET report date preceded date of last DFCI clinic visit) for 344 of 506 (68%) patients. If a clinical trial was recommended and the report was e-mailed to the primary oncologist before the trial consent date, this was considered a GI TARGET–associated enrollment. If on- or off-label therapy was recommended and the report was e-mailed to the primary oncologist before treatment initiation, this was counted as a GI TARGET–associated action. If additional testing was recommended and the report was e-mailed to the primary oncologist before placement of the testing order, this was counted as a GI TARGET–associated action. If a referral to the Dana-Farber Genetics and Prevention clinic was made after a report that included a recommendation for germline genetic evaluation was e-mailed to the primary oncologist, this was also considered a GI TARGET–associated action.

## RESULTS

To determine the scope and scalability of the GI TARGET program, we assessed the review process for the retrospective cohort including descriptive statistics of our patient population, the volume of cases reviewed, the effort required for and efficiency of review, and characterization of recommendations made in reports. We also sought to assess the impact of the program on patients with GI cancer by determining clinical follow-up on recommendations where data were available.

### Programmatic Review of Tumor Molecular Profiling

**FIG 3.**
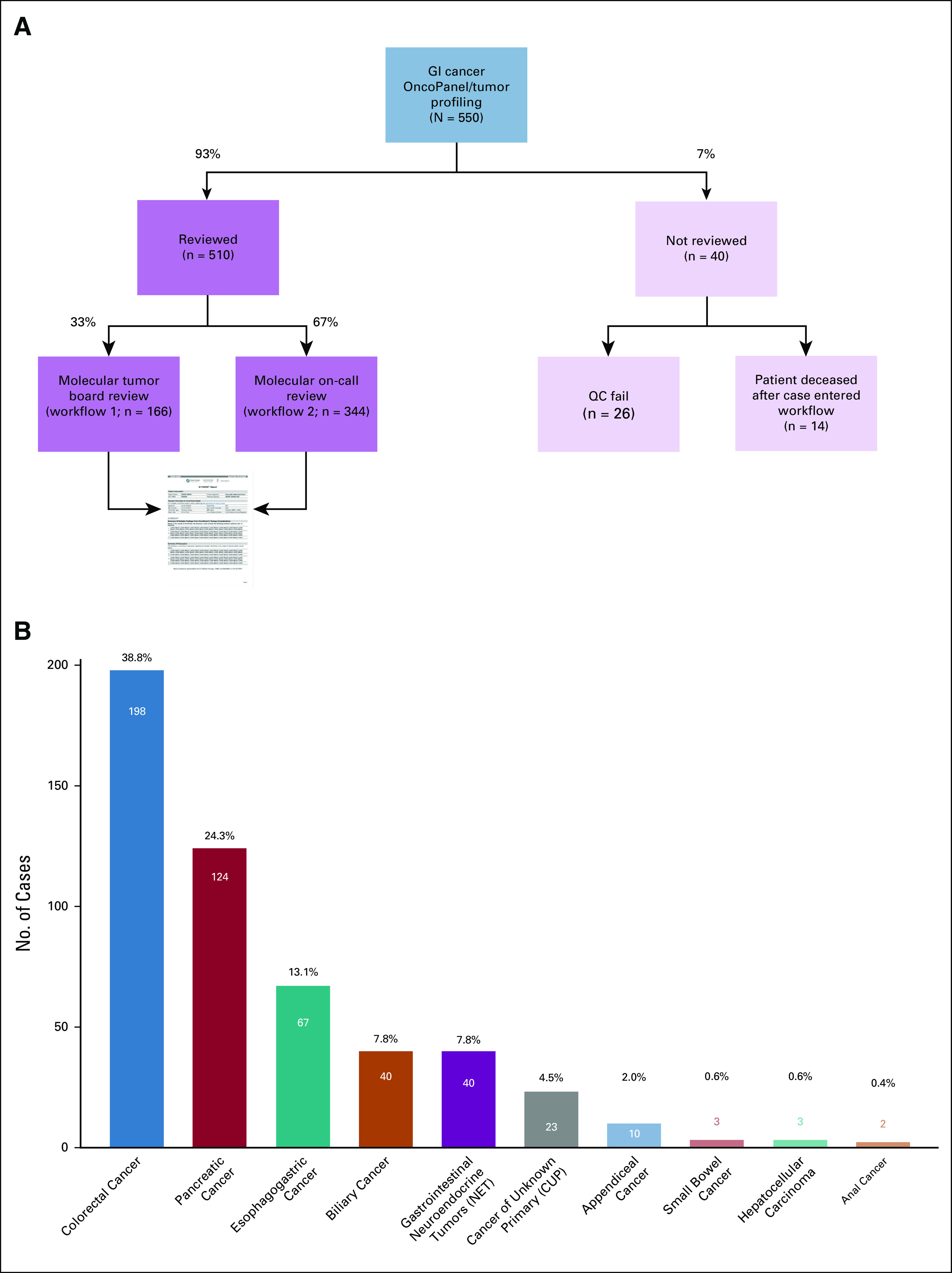
GI TARGET retrospective cohort: (A) case review flowchart and (B) cancer types in cohort. The number of cases and percentage of total cases (N = 510) for each cancer type are presented above each bar. GI TARGET, Gastrointestinal Treatment Assistance Regarding Genomic Evaluation of Tumors.

GI TARGET reports were generated on 93% of tumor profiling results (510 of 550) corresponding to 93% of patients (506 of 542; Fig 3A). The predominant cancer types reviewed included colorectal (n = 198, 39%), pancreatic (n = 124, 24%), esophagogastric (n = 67, 13%), biliary cancers (n = 40, 8%), GI neuroendocrine tumors (n = 40, 8%) and cancers of unknown primary at the time of review (n = 23, 5%; Fig 3B). Selected clinical data for this cohort are presented in Supplemental Tables 2-6 in the Data Supplement. Of note, GI stromal tumors are treated in the Sarcoma Center at DFCI and are thus not included in our cohort. 33% of cases (166 of 510) were reviewed via Workflow 1 in the weekly MTB, and 67% of cases (344 of 510) were reviewed via Workflow 2 using the Molecular On-Call system (Fig 3A). An average of 20 (range 8-42) cases were received for review per week. Of these, an average of six (range 1-15) cases were triaged to Workflow 1, and an average of 13 (range 0-27) cases were triaged to Workflow 2 (Supplemental Figure 2A in Data Supplement). The median turnaround time (TAT) for reports reviewed through Workflow 1 was 4 (range 0-28) days, whereas the median TAT for reports reviewed through Workflow 2 was 9 (range 4-23) days. The median overall TAT for GI TARGET reports was 8 (range 0-28) days (Supplemental Figure 2B in Data Supplement). The data transfer time—from OncoPanel sign-out to when the data are ingested into MatchMiner and algorithmically matched—is 1-2 days. Therefore, the time between provider receipt of the OncoPanel report and the GI TARGET report is estimated to be 9-10 days. We estimate that the combined effort required to prepare a single report—from case triaging through review and report finalization—is 1.9 person-hours. In an average week reviewing 20 cases, this equates to 38 person-hours of combined GI TARGET team effort. Furthermore, 1.0 Full-Time Equivalent of a PhD-level CGS was required to review all OncoPanels from GCC patients on a rolling basis in addition to overall program management. Workflow adjustments have since been implemented to maximize utility (Data Supplement).

### Molecularly Guided Clinical Recommendations

90% of reports (460 of 510) included ≥ 1 recommendation for the treating oncologist (mean 3, range 0-11). A summary of recommendations made for the 506 patients in the retrospective cohort is presented in Table 1, and a report-by-report accounting of recommendations is presented in Supplemental Figure 3 in the Data Supplement. 81% of patients (412 of 506) were recommended treatment with one or more molecularly guided therapies: 80% (406 of 506) were recommended enrollment on one or more precision medicine clinical trial(s) with an average of two clinical trial recommendations (range 0-9) per report, 6% (28 of 506) were recommended on-label treatment with a molecularly targeted agent, and 25% (125 of 506) were recommended off-label treatment with a molecularly targeted agent. Additional or orthogonal molecular testing was recommended for 42% of patients (211 of 506): 31% (158 of 506) for germline genetic evaluation (eg, if the patient was young at initial diagnosis or if a suspected germline alteration was detected on OncoPanel), 3% (13 of 506) for immunohistochemistry staining of tumor tissue (eg, mismatch repair, human epidermal growth factor receptor 2, and programmed death-ligand 1), 3% (15 of 506) for RNA-based fusion detection (eg, to explore the functional effect of a detected rearrangement), and 13% (65 of 506) for repeat OncoPanel testing (eg, if the sample sequenced poorly or if an old sample was sequenced and recent disease progression was noted in the EHR, suggesting possible tumor evolution). The frequency of recommendations included in GI TARGET reports differed between cancer types consistent with clinical practice, current knowledge of GI cancer molecular landscapes, and available therapeutic options (Table 2 and Data Supplement). Example recommendations and comments for a case of pancreatic cancer are presented in Figure 2B.

**TABLE 1.**
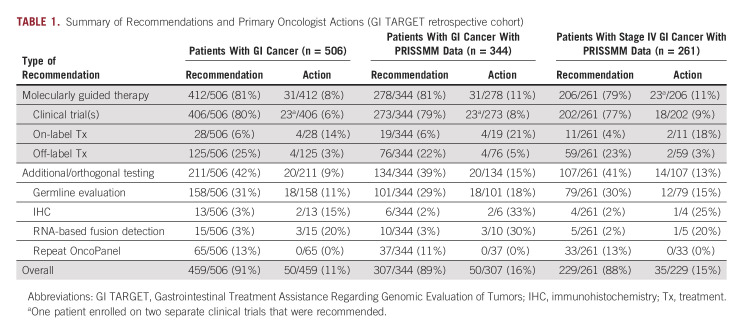
Summary of Recommendations and Primary Oncologist Actions (GI TARGET retrospective cohort)

**TABLE 2.**
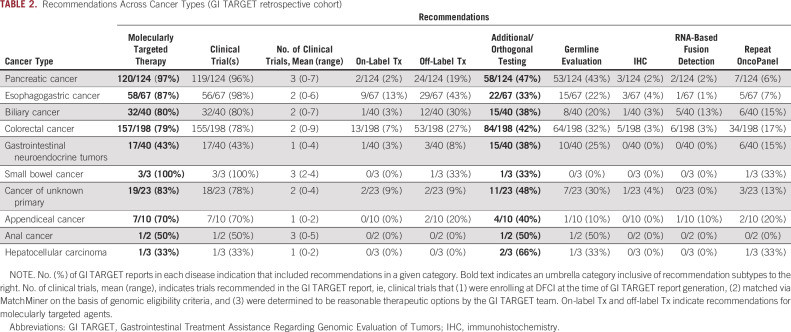
Recommendations Across Cancer Types (GI TARGET retrospective cohort)

### Impact on Clinical Care Decision Making

**FIG 4.**
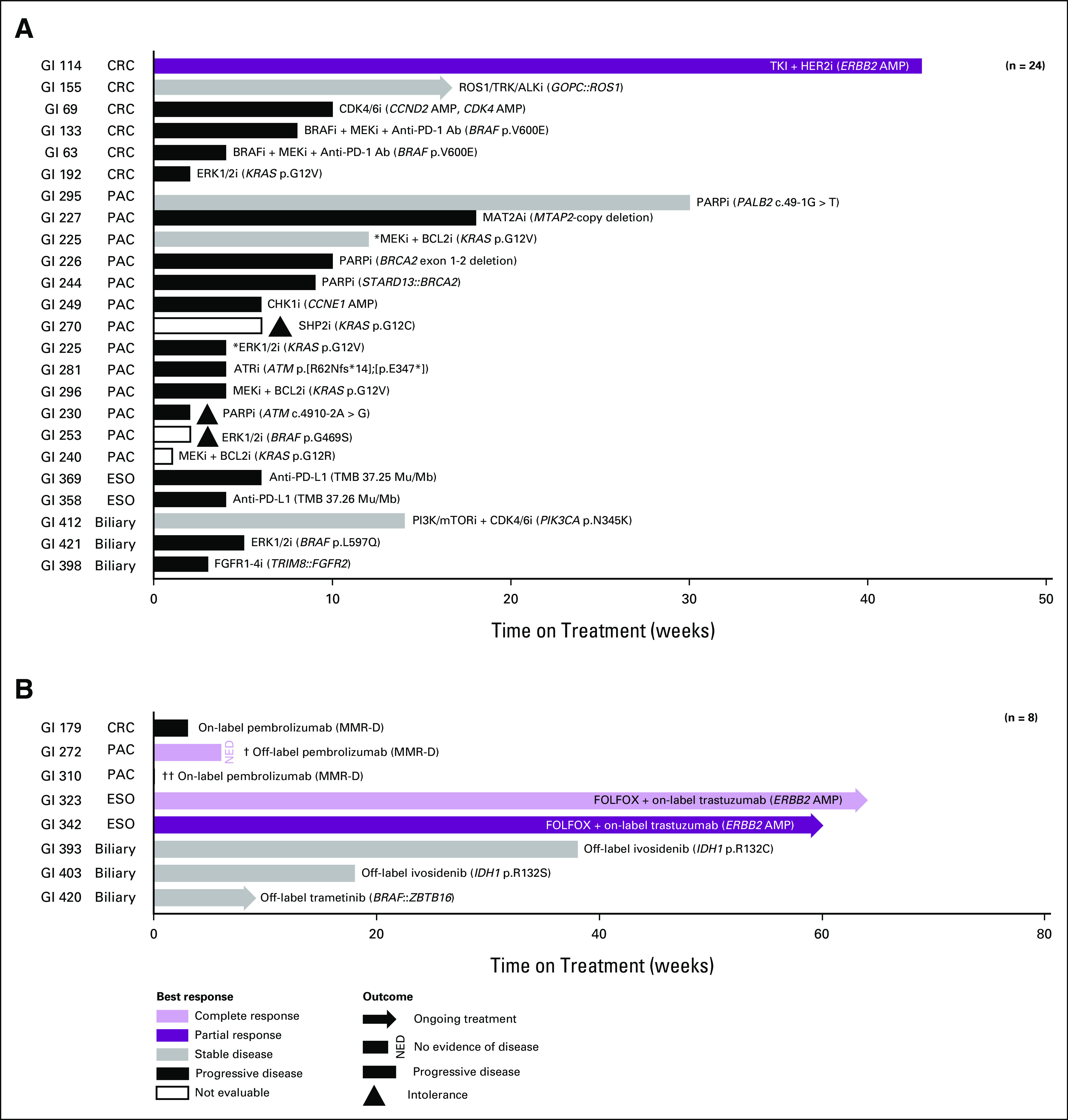
Molecularly targeted therapy on clinical trials/on-label/off-label (GI TARGET retrospective cohort): (A) best response and time on treatment for 24 clinical trial enrollments associated with GI TARGET recommendations in the retrospective cohort. Toxicity/intolerance/adverse event are only noted if these contributed to the patient discontinuing treatment. An asterisk (*) denotes enrollment of a patient on two separate clinical trials (4th and 5th lines of therapy, respectively) on the basis of the same alteration (*KRAS* p.G12V). (B) Best response and time on treatment for eight patients receiving on- and off-label molecularly targeted therapy in the retrospective cohort. Toxicity/intolerance/adverse event are only noted if these contributed to the patient discontinuing treatment. A dagger (†) denotes a patient who received both neoadjuvant and adjuvant treatment. Two daggers (††) denote a patient who was on hospice before treatment start and elected to return to hospice because of worsening symptoms immediately after treatment start. AMP, amplification; CRC, colorectal cancer; ESO, esophagogastric cancer; FOLFOX, folinic acid + fluorouracil + oxaliplatin; ESO, esophagogastric cancer; GI TARGET, Gastrointestinal Treatment Assistance Regarding Genomic Evaluation of Tumors; MMR-D, mismatch repair deficiency; NED, no evidence of disease; PAC, pancreatic cancer.

The primary oncologist took clinical action consistent with recommendations in GI TARGET reports for 16% of patients with evaluable clinical follow-up (50 of 307; Table 1). We tracked 24 clinical trial enrollments (involving 23 patients) that were associated with GI TARGET recommendations in the retrospective cohort (Fig 4A, and Supplemental Figures 3 and 7 in Data Supplement). 71% (17 of 24) of enrollments were on a specific protocol that was recommended, whereas 29% (7 of 24) of enrollments were on an alternative protocol with a suggested targeted agent or a class of agents or based on an alteration that was highlighted in the GI TARGET report. Of note, a single patient was enrolled on two clinical trials that were recommended on the basis of the same alteration (*KRAS* p.G12V). Moreover, 21% of patients (4 of 19) underwent treatment with an US Food and Drug Administration–approved targeted therapy that was recommended, 5% (4 of 76) of patients received off-label targeted therapy that was recommended (Fig 4B; and Supplemental Figure 3 and Supplemental Table 8 in Data Supplement), and 15% (20 of 134) of nontherapeutic recommendations were acted upon (Table 1 and Supplemental Figure 3 in Data Supplement), suggesting the impact of GI TARGET on patient care beyond the choice of targeted therapy. Notably, identification of potential germline alterations as secondary findings of tumor profiling is well recognized and guidance has been suggested regarding follow-up.^16,17^ The most potentially impactful aspect of germline referrals arising from GI TARGET likely stems from referral of patients who would not otherwise be considered for germline evaluation, eg, based on personal or family history (Supplemental Figure 4 and Supplemental Data in Data Supplement).

## DISCUSSION

We developed a system for programmatic review of tumor molecular testing to inform clinical decision making within the DFCI GCC. Our program adds value to OncoPanel and other tumor profiling through computationally driven clinical trial matching, multidisciplinary review of tumor genomics, and prioritization of therapeutic options. Here, we present a retrospective analysis of genomics review for 506 patients with GI cancer who underwent molecular testing between January and June 2019 with demonstrated utility for informing patient care. The program is scalable and sustainable as evidenced by review of > 2,700 cases since launching in 2018, and the utility of the program is evidenced by various forms of provider engagement (Data Supplement). Clinical action on the basis of the tumor molecular profile was suggested for 91% of patients: for 81% of patients, recommendations were made for targeted therapy whether on a clinical trial or on- or off-label; for 42% of patients, additional molecular testing was recommended. A number of patients benefited as evidenced by clinical provider action after release of GI TARGET reports. Although provider actions were undoubtedly influenced by multiple factors, the objective of the GI TARGET program is to support provider decision making by offering additional and/or alternative analysis of tumor profiling and associated precision oncology options and is intended to be considered together with other knowledge, expertise, and resources. While the rate of clinical action on the basis of recommendations was modest in our cohort (16% of patients for whom follow-up data were available), this number should be interpreted in the context of short follow-up and universal review of cases since the trigger for GI TARGET review is the release of OncoPanel results as opposed to a change in clinical status or provider request. To extend the utility of the GI TARGET reports beyond the timeframe immediately after MTB review, recommendations were written to highlight potentially targetable genes or pathways on the basis of actionable alterations, whereas available clinical trials and approved therapies were presented as current options. We also highly encourage our oncologists to routinely check MatchMiner for up-to-date genomically driven trial options for their patients and to request updated GI TARGET review as needed.

Enrollment on clinical trials in the United States is estimated at just 2%-3% of adult patients with cancer.^18,19^ Low clinical trial enrollment makes reaching study accrual goals difficult and may limit advances in patient care. Moreover, this suggests that many patients with cancer either do not have access to or else do not take advantage of additional lines of therapy after standard-of-care options have been exhausted. Several studies have attempted to identify barriers to clinical trial enrollment.^19,20^ Unger et al^19^ classify these into Barrier Domains: Structural (ie, availability of trials), Clinical (ie, eligibility for trials), Physician (ie, offer and discussion of clinical trial options), and Patient (ie, personal factors affecting the decision to enroll). Both MatchMiner and GI TARGET were developed to address obstacles in the Physician and Patient domains by streamlining the assessment of clinical trial eligibility and by facilitating and encouraging oncologists to discuss trial options with their patients, a key point of intercession given that oncologists are a principal source of clinical trial information.^21^

We were able to identify clinical trial enrollments informed by GI TARGET recommendations in our cohort in a little more than 8% (23 of 273) of patients for whom a trial was recommended. This rate was slightly higher in stage IV patients at 9% (18 of 202). We believe this rate reflects the minimum impact that GI TARGET and MatchMiner could have on clinical trial enrollment for these patients given that (1) reports were automatically and universally generated for all patients with OncoPanel testing regardless of the current line of therapy and response to or progression on current therapy and irrespective of whether the patient was clinically or logistically a trial candidate, (2) therapeutic recommendations were made regardless of the cancer stage so as to be useful in the case of future disease progression or recurrence, (3) the follow-up time for our retrospective analysis was short such that some patients may yet enroll on a recommended trial at DFCI, and (4) our accounting does not include enrollment on clinical trials at other centers. Although it is very important to understand the contribution of these and other factors to trial nonenrollment, a limitation of our study is that it was not designed to assess the reason(s) for nonenrollment on a patient-by-patient basis.

While the promise of precision oncology is yet to be fully realized,^22^ it is increasingly regarded as another tool of the trade.^23^ We have described in detail an example program for the systematic assessment of molecularly guided treatment and clinical care options for patients with cancer on the basis of tumor profiling. GI TARGET was developed with the resources of a large academic center, which included the committed effort of clinicians and researchers and data science and software engineering support. As many of these resources primarily serve to optimize logistics, similar programs could be developed in centers with smaller case volumes, such as community hospitals, or by establishing request-only precision oncology tumor boards. Additional considerations in community health care settings will include the availability of trials and multidisciplinary expertise, which will present distinct challenges compared with the academic setting. Nevertheless, it is our hope that the GI TARGET program can be instructive toward the development of these programs, either as standalone efforts or in collaboration with academic centers, and that our experience can inform the universal implementation of precision oncology.
